# Supplementary material for: Decoding protein methylation function with thermal stability analysis
Source: Nat Commun. 2023 May 25;14:3016. doi: 10.1038/s41467-023-38863-1 (PMC10212966; doi:10.1038/s41467-023-38863-1)
Supplement: Supplementary file 1 — Supplementary Information [file 41467_2023_38863_MOESM1_ESM.pdf]

Supplementary material for:

DECODING PROTEIN METHYLATION FUNCTION WITH THERMAL STABILITY ANALYSIS

Cristina Sayago[1], Jana Sánchez-Wandelmer[1], Fernando Garcia[1], Begoña Hurtado[2,3], Vanesa Lafarga[4], Patricia Prieto[5], Eduardo Zarzuela[1], Pilar Ximénez-Embún[1], Sagrario Ortega[5], Diego Megias[6], Oscar Fernández-Capetillo[4], Marcos Malumbres[2,3,7], Javier Munoz[1,8,9]

[1] Proteomics Unit, Spanish National Cancer Research Centre (CNIO), 28029 Madrid (Spain)
[2] Cell Division and Cancer Group, Spanish National Cancer Research Centre (CNIO), 28029 Madrid (Spain)
[3]Cancer Cell Cycle group, Vall d'Hebron Institute of Oncology (VHIO), 08035 Barcelona, (Spain)
[4] Genomic Instability Group, Spanish National Cancer Research Centre (CNIO), 28029 Madrid (Spain)
[5] Mouse Genome Editing Unit, Spanish National Cancer Research Centre (CNIO), 28029 Madrid (Spain)
[6] Confocal Microscopy Unit, Spanish National Cancer Research Centre (CNIO), 28029 Madrid (Spain)
[7]Catalan Institution for Research and Advanced Studies (ICREA), 08010 Barcelona, (Spain)
[8] Cell Signaling and Clinical Proteomics Group, Biocruces Bizkaia Health Research Institute, 48903 Barakaldo (Spain)
[9] Ikerbasque, Basque foundation for science, 48011. Bilbao (Spain)

Correspondence to: javier.munozperalta@osakidetza.eus

- Supplementary Figures 1-22
- Supplementary References

SUPPLEMENTARY FIGURES

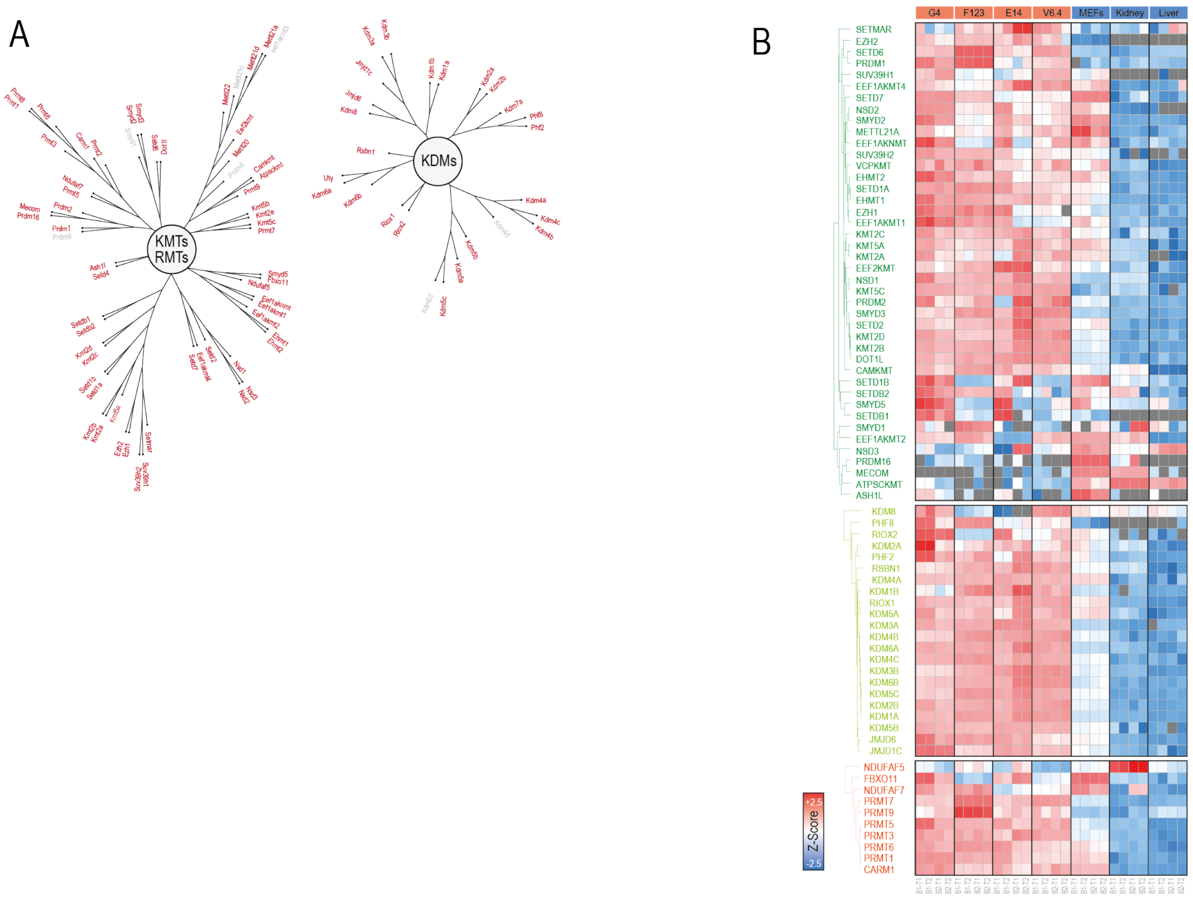

Supplementary Figure 1. Mass spectrometric characterization of the mESCs and MEFs proteomes. A, Phylogenetic tree of all methyl-transferases (lysine and arginine) (left) and de-methylases (lysine) (right) annotated in Uniprot. Identified enzymes in the current data are shown in red. B, heatmaps representing the relative intensities (z-score) of KMTs (dark green), KDMs (light green) and RMTs (orange) quantified by PRM in four mES cell lines (G4, F123, E14 and V6.4), MEFs, and two mouse tissues (kidney and liver). Two biological replicates (B1, B2) and two technical replicates (T1, T2) were analysed.

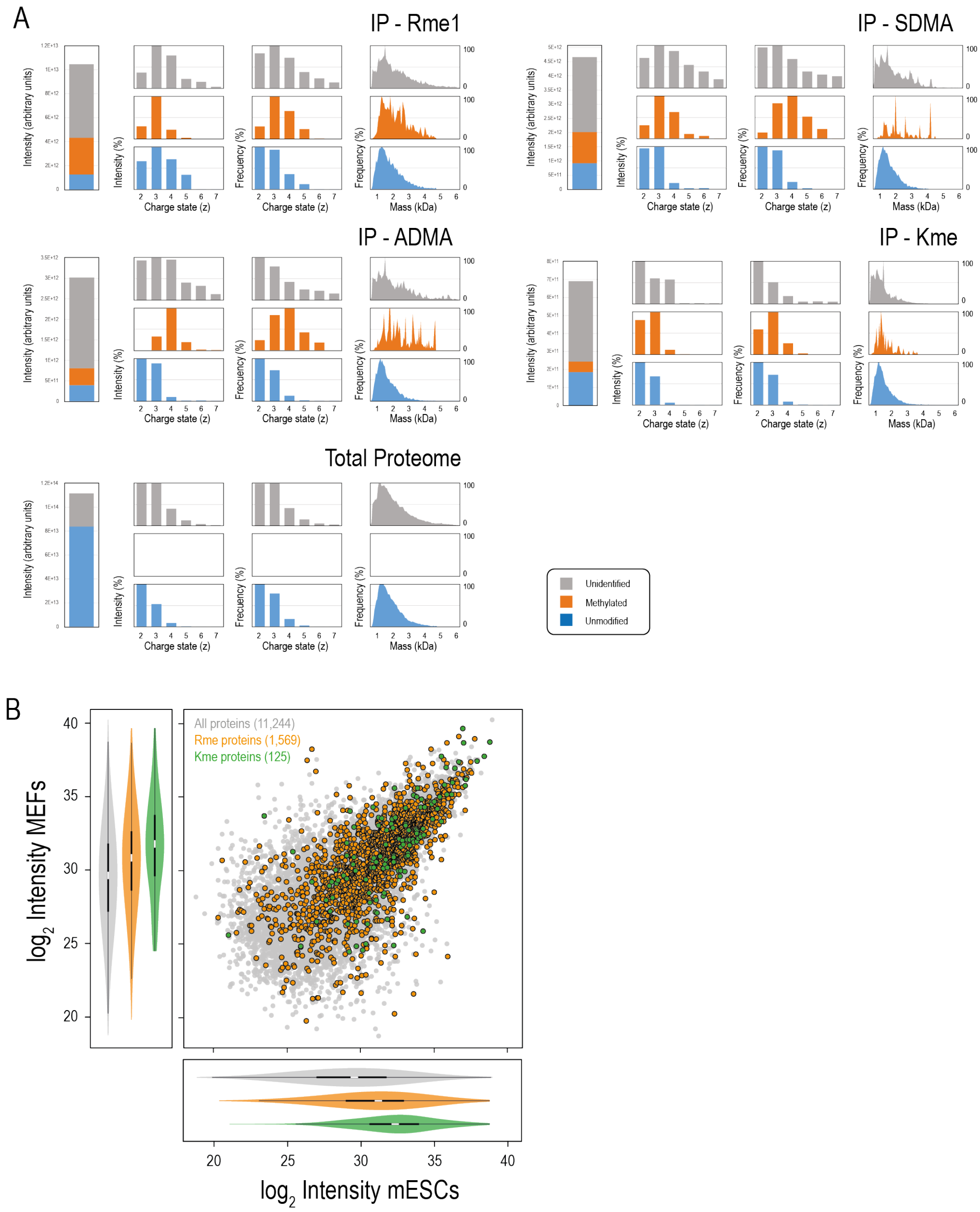

Supplementary Figure 2. Under-sampling of methyl-proteomes. A, Intensities (arbitrary units) of identified (methylated or unmodified) peptides as well as unidentified precursor ions detected in each of the four immunoprecipitations (left). On the right, the histograms of charge states (showing the total intensity and the frequency) well as mass of precursor ions. As a reference, the distributions of identified an unidentified peptides found in the complete mESCs proteome data set (Suppl Table 1) are shown. B, Scatterplot comparing protein intensities between mESCs and MEFs. Proteins found methylated in Arg and Lys residues in our immune-purifications are highlighted in colors and their distributions are shown in violin plots in both axes. For violin plots, median is shown; box limits indicate the 25th and 75th percentiles; whiskers extend 1.5 times the interquartile range from the 25th and 75th percentiles. Source data are provided as a Source Data file.

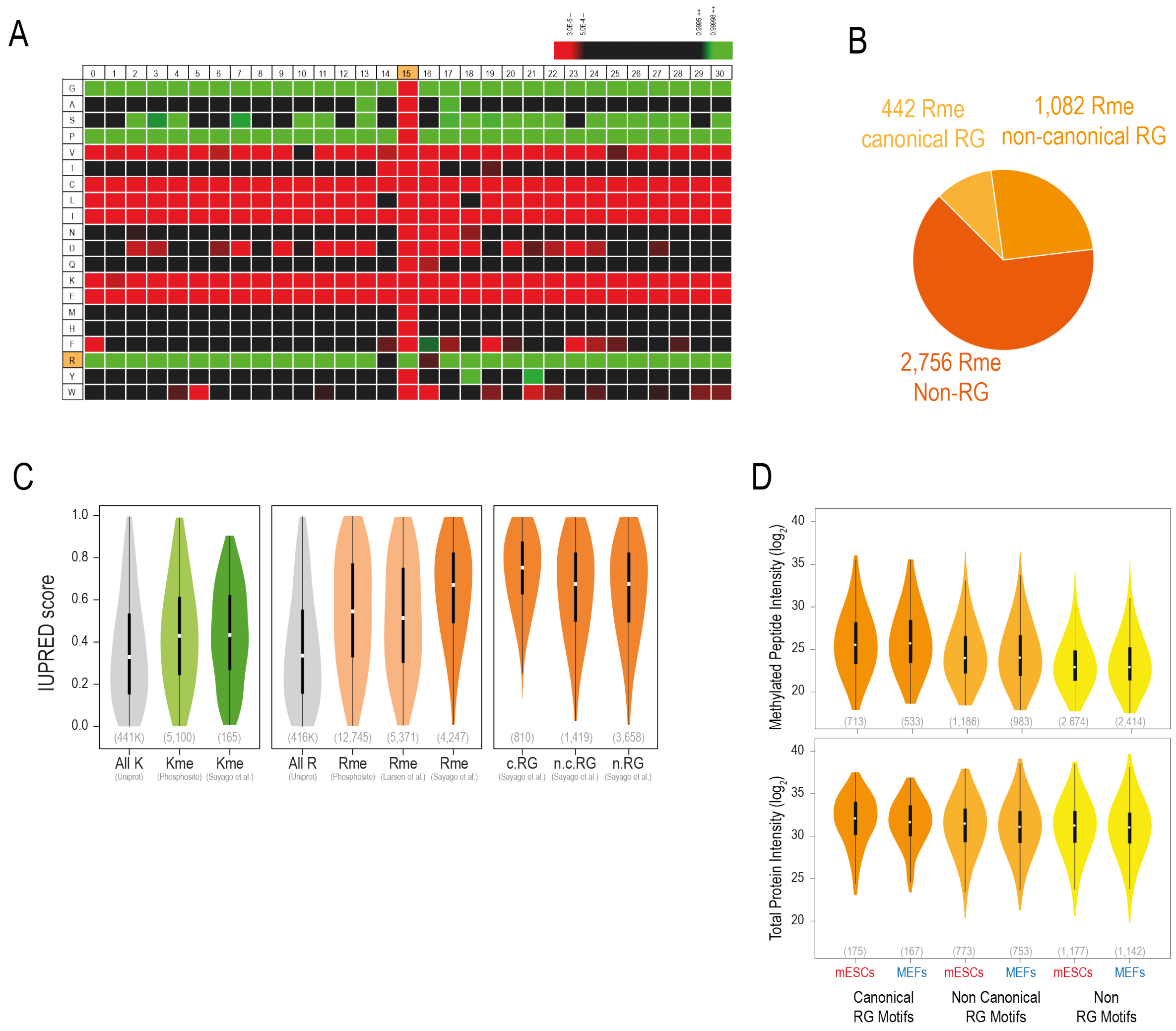

Supplementary Figure 3. Sequence analysis of identified methylated peptides. A, Sequence window containing +- 15 residues upstream or downstream the Arg methylated sites were analyzed with IceLogo to find over-represented amino acids along the identified methylated peptides. B, pie chart showing the number of Arg sites classified as canonical RG, non-canonical RG and non-RG motifs. C, Violin plots showing the distribution of IUPRED scores (indicative of the probability of a residue being part in a low complexity region) for the identified Lys and Arg methylated residues in the current work (Sayago *et al.*). Additional data sets are also shown for comparison (Phosphosite and Larsen *et al.*[1]), and as a refence, all Arg and Lys residues in the mouse proteome are included. D, Distribution of the intensities of methylated (upper) and total proteins (bottom) classified according to their corresponding Arg motif, which shows that canonical RG sites tend to have a higher stoichiometry than the other sites. For violin plots, median is shown; box limits indicate the 25th and 75th percentiles; whiskers extend 1.5 times the interquartile range from the 25th and 75th percentiles. Sample size in each category is shown below in parenthesis. Source data are provided as a Source Data file.

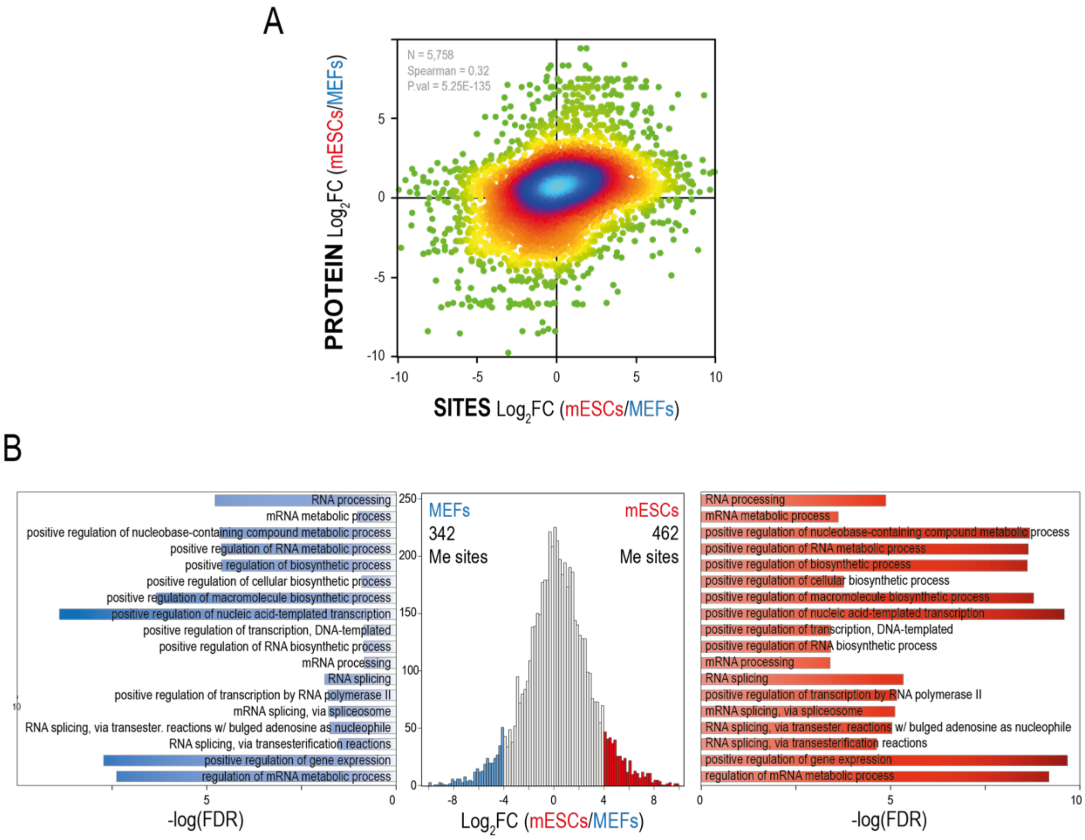

Supplementary Figure 4. Comparison of methylation levels between mESCs and MEFs. A, Log2 ratios (mESCs/MEFs) were calculated for 5,758 sites and compared to their corresponding protein log2 ratios. Spearman's rank correlation coefficient and its associated p.value were calculated in Perseus and are shown in the figure. The color scale reflects the density of points. B, histogram showing the distribution of log2 ratios between mESCs and MEFs for all the identified Arg and Lys methylated sites. Ratios above 4 or below -4 are considered enriched in mESCs and MEFs respectively. GO biological process enriched in each case are shown.

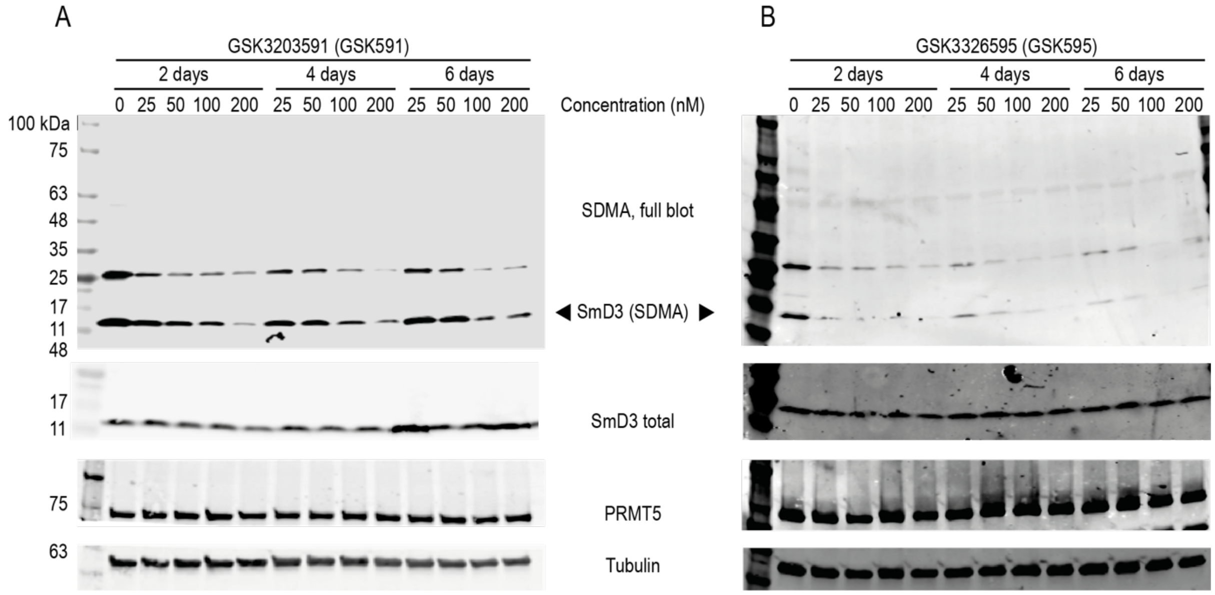

Supplementary Figure 5. Symmetric di-methylation levels of Snrpd3 in response to Prmt5 inhibitors in mESCs. mESCs were treated with DMSO or with two Prmt5 inhibitors: GSK3203591 (GSK591) (A) and GSK3326595 (GSK595) (B) for different time points (2, 4, 6 days) and concentrations (0, 25, 50, 100, 200 nM). Samples (one biological replicate) were analyzed by western blot using a pan-specific antibody against symmetric Arg methylation (SDMA). The band detected at 13 kDa corresponds to Snrpd3 (SmD3)[2]. Total Snrpd3 protein levels were also assessed and no major variations were found. Prmt5 protein levels were also measured and no major variations were found. Tubulin levels are presented as loading control. This experimented was repeated twice. Source data are provided as a Source Data file.

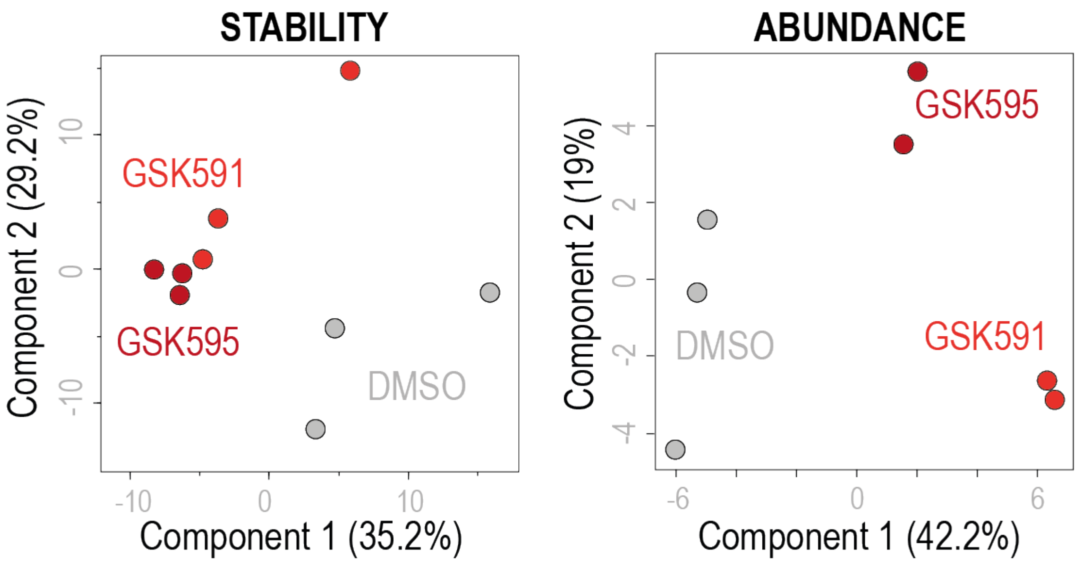

Supplementary Figure 6. Principal component analysis of stability and abundance changes upon Prmt5 inhibition in mESCs. Stability and abundance changes for all identified proteins in mESCs were analyzed in Perseus using Principal Component Analysis. Each dot represents a biological replicate.

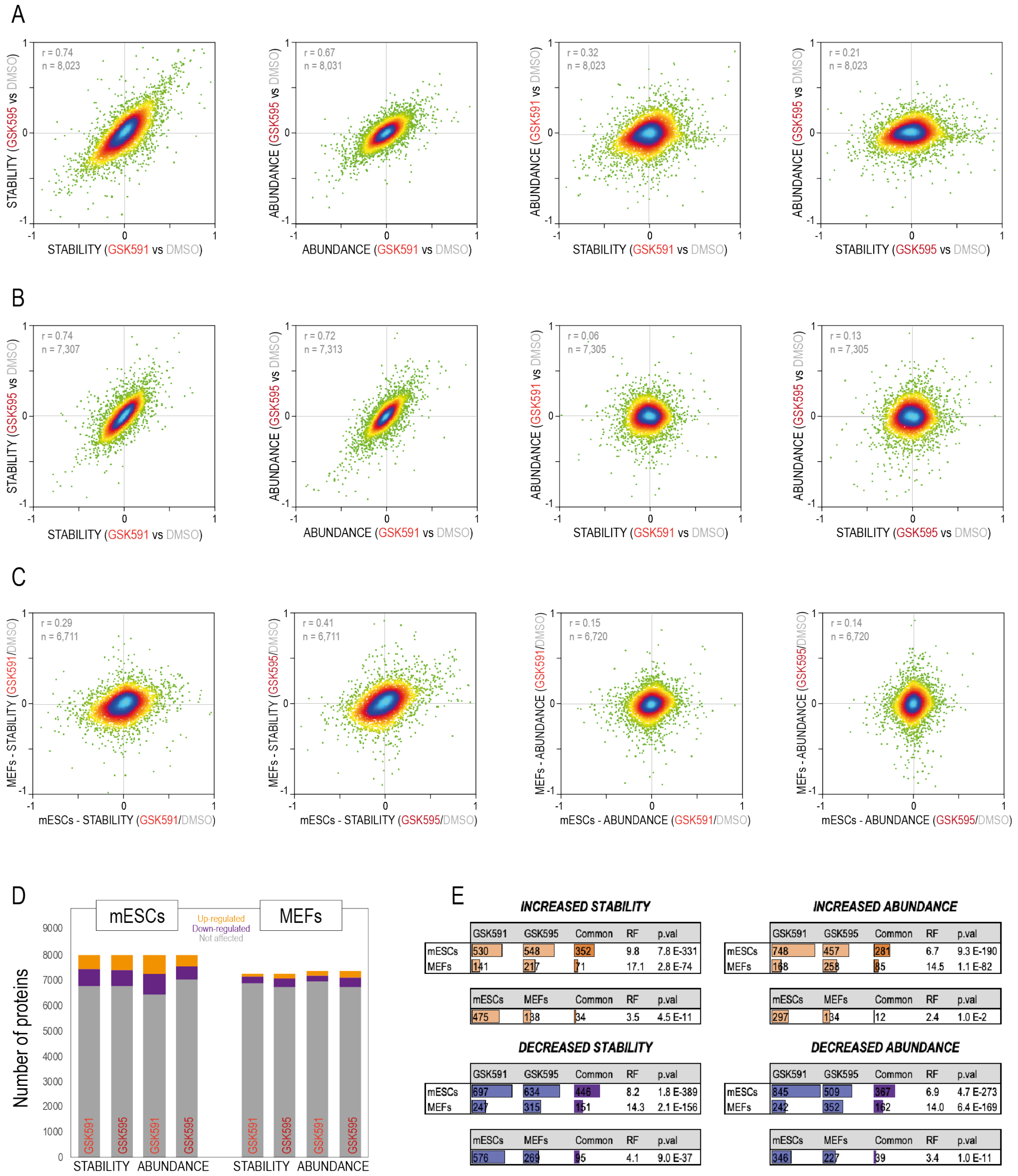

Supplementary Figure 7. Thermal stability and protein abundance levels in mESCs and MEFs upon Prmt5 inhibition. A, density scatterplots comparing stability and abundance changes in mESCs. B, density scatterplots comparing stability and abundance changes in MEFs. A, density scatterplots comparing stability and abundance changes between mESCs and MEFs. D, number of up- and down-regulated proteins (moderated t-test *limma* two-sided, FDR <5%) found in each comparison. E, number of proteins that were found regulated in common between both compounds (up) and between mESCs and MEFs (down) for stability and abundance changes. The significance of the overlap (p.val) in each comparison was calculated with the hypergeometric test (two-sided). RF, representation factor. Source data are provided as a Source Data file.

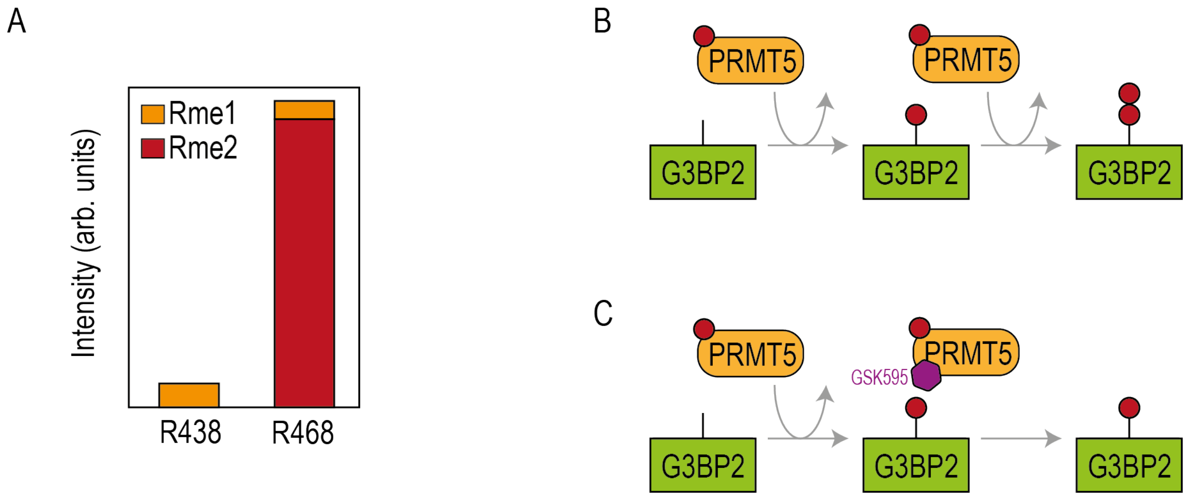

Supplementary Figure 8. Prmt5 di-methylates Grbp2 at position R468 in a distributive manner. A, The intensity (arb. units, arbitrary units) of mono- and di-methylation identified in R438 and R468 of G3bp2 in the *in vitro* methyl-transferase assay of Prmt5 is presented. Average of two biological replicates and two technical replicates. B, Prmt5 methylates protein substrates in a distributive manner. C, GSK595, a substrate competitive Prmt5 inhibitor, prevents its binding to the mono-methylated substrate and thereby the subsequent addition of a second methyl group.

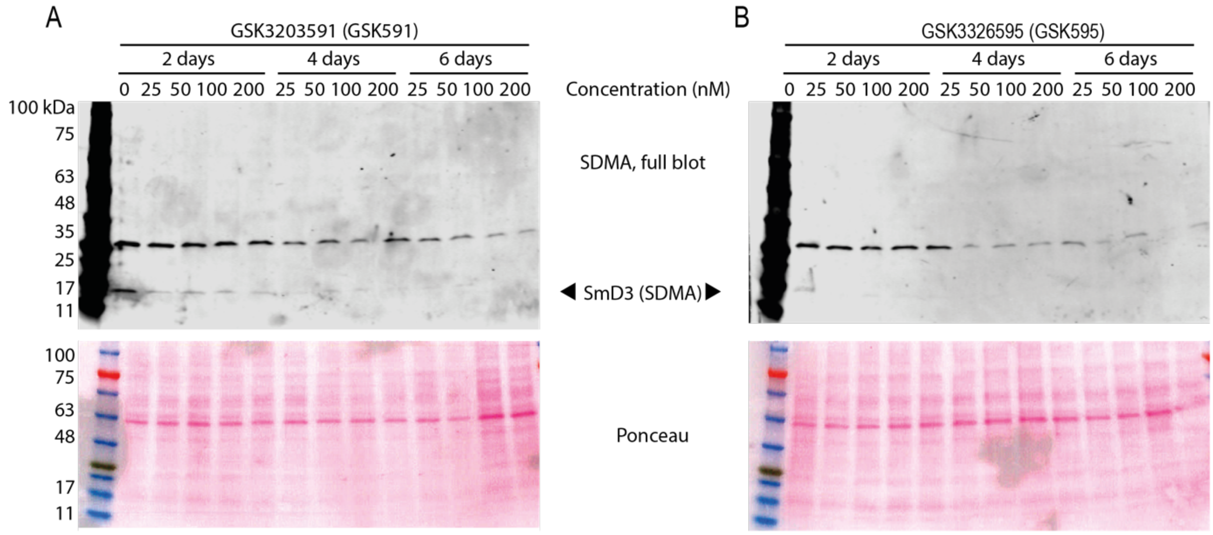

Supplementary Figure 9. Symmetric di-methylation levels of Snrpd3 in response to Prmt5 inhibitors in MEFs. MEFs were treated with DMSO or with two Prmt5 inhibitors: GSK3203591 (GSK591) (A) and GSK3326595 (GSK595) (B) for different time points (2, 4, 6 days) and concentrations (0, 25, 50, 100, 200 nM). Samples (one biological replicate) were analyzed by western blot using a pan-specific antibody against Arg methylation. The band detected at 13 kDa corresponds to Snrpd3 (SmD3) (uncropped image)[2]. Membrane (red Ponceau) is shown as a loading control. This experiment was repeated twice.

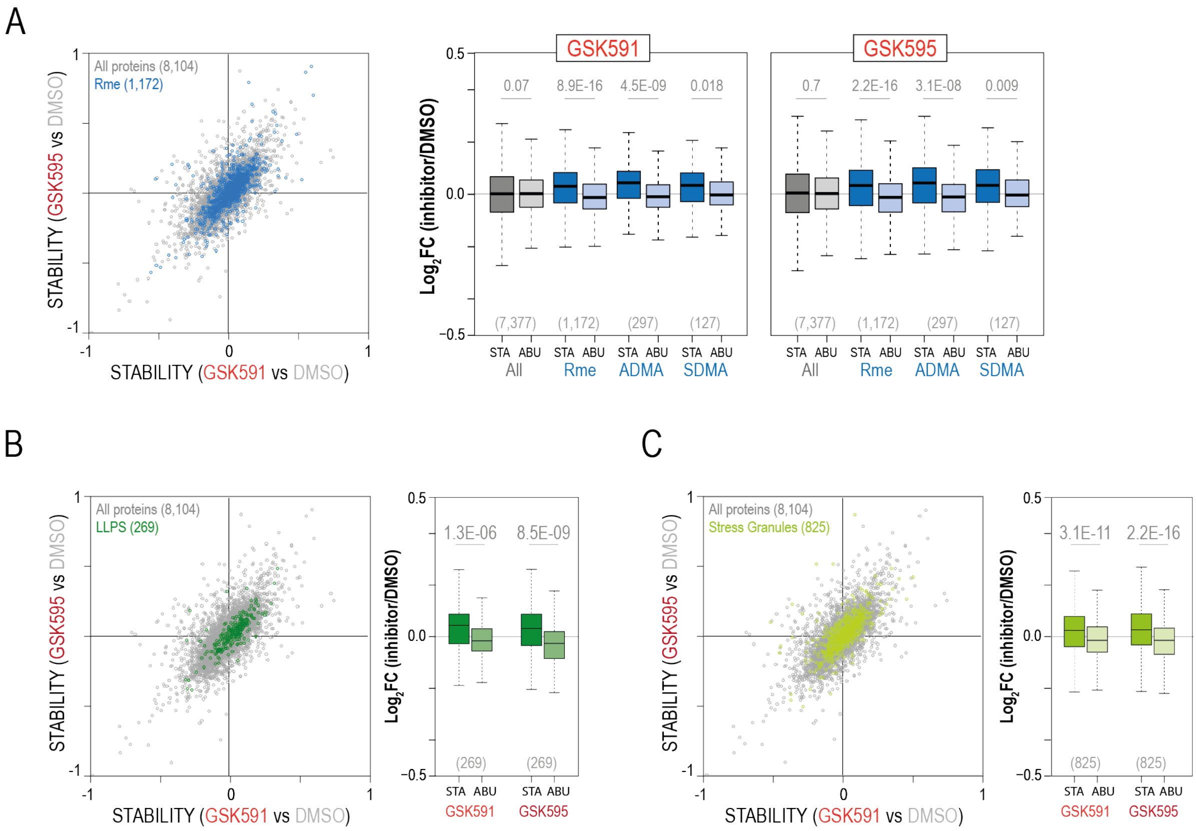

Supplementary Figure 10. Thermal stability and abundance changes upon Prmt5 inhibition in MEFs. A, Box-plots show the distributions of Stability (S) and Abundance (A) log2 ratios for all identified proteins as well as for Arg methylated proteins, asymmetric-dimethylated proteins and symmetric-dimethylated proteins identified in our data. The scatterplot on the left shows the actual distribution for all Arg methylated proteins identified in our data. B, Scatterplot and box-plots showing the distributions of Stability (S) and Abundance (A) log2 ratios for all proteins annotated as regulated by liquid-liquid phase separation in Uniprot. C, Scatterplot and box-plots showing the distributions of Stability (S) and Abundance (A) log2 ratios for all proteins annotated as being part or associated to stress granules in two different publications[3,4]. For box-plots, median is shown; box limits indicate the 25th and 75th percentiles; whiskers extend 1.5 times the interquartile range from the 25th and 75th percentiles. Sample size in each category is shown below in parenthesis. P-values (A-C) are calculated using a non-parametric Wilcoxon test (two-sided). Source data are provided as a Source Data file.

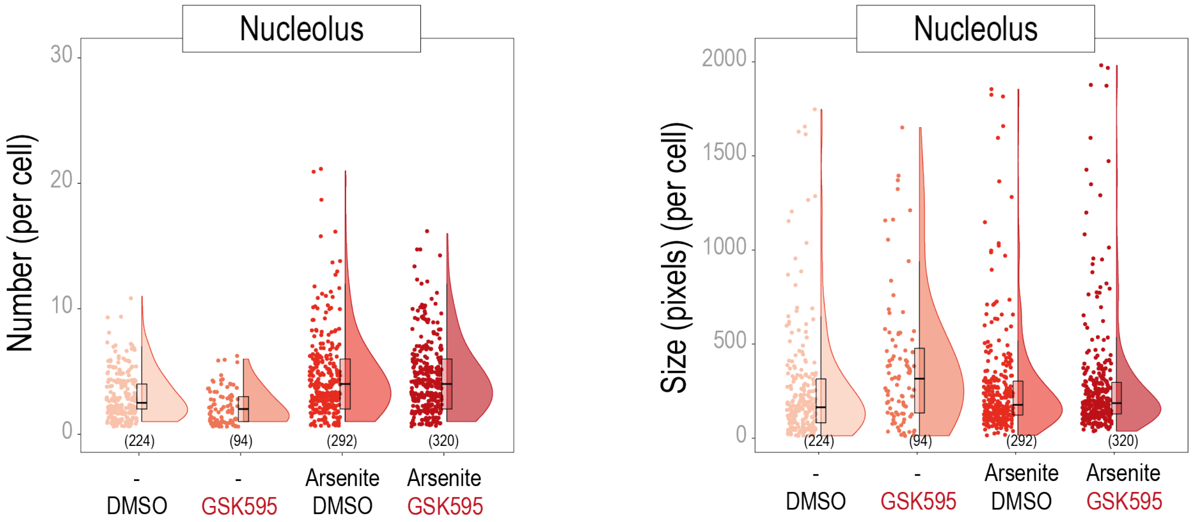

Supplementary Figure 11. Quantification of confocal microscopy images to assess the fragmentation of nucleolus. The violin plots show the number of nucleoli per cell (left) as well as the average size (right) in response to the indicated conditions (below). For box-plots, median is shown; box limits indicate the 25th and 75th percentiles; whiskers extend 1.5 times the interquartile range from the 25th and 75th percentiles. Sample size (independent measurements) in each condition (n) is shown below in parenthesis. Source data are provided as a Source Data file.

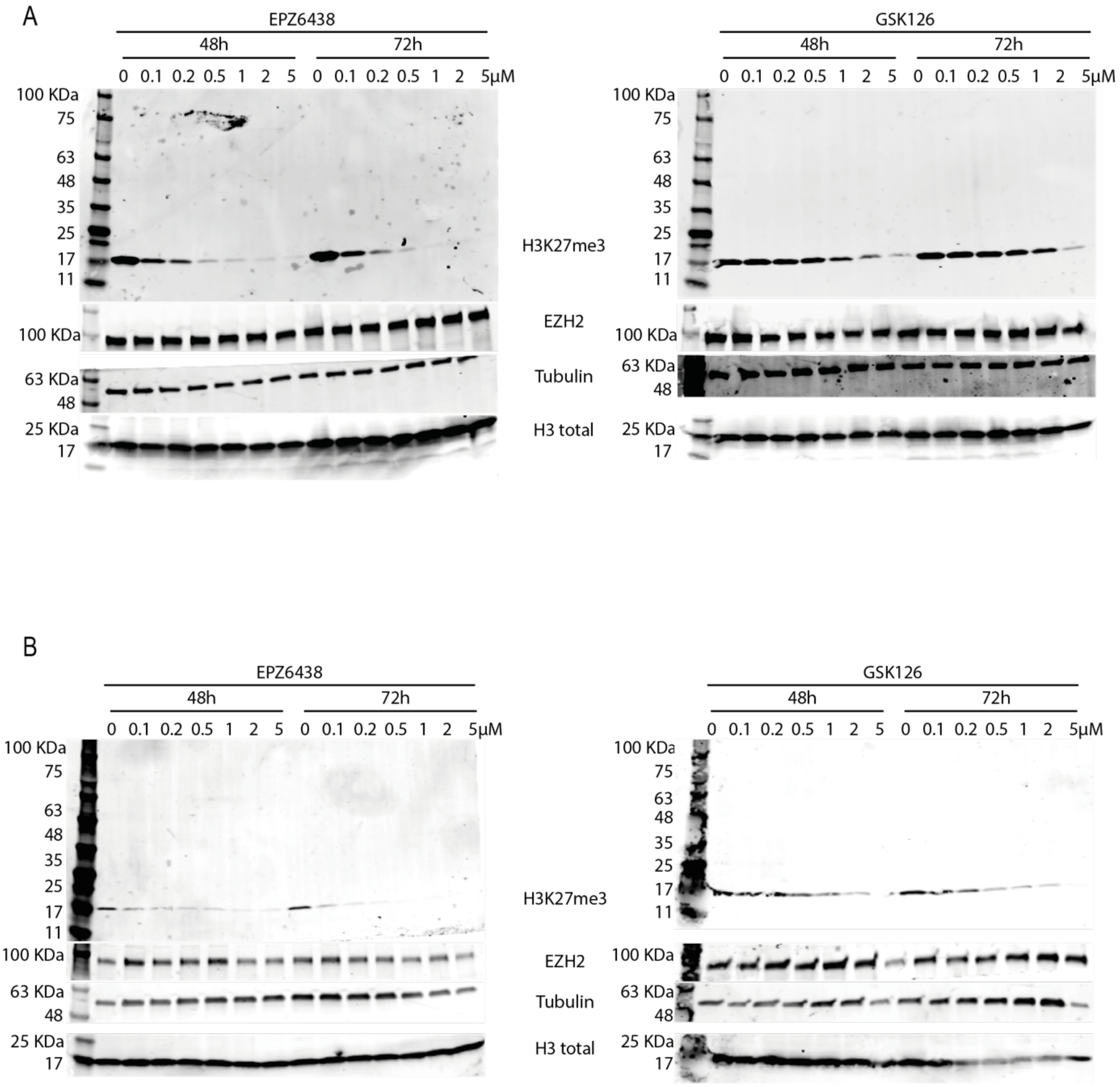

Supplementary Figure 12. H3K27me3 levels in response to two different Ezh2 inhibitors in mESCs and MEFs. mESCs (A) and MEFs (B) were treated with DMSO or with two Ezh2 inhibitors: EPZ6438 and GSK126 for different time points (2, 3 days) and concentrations (0, 0.1, 0.2, 0.5, 1, 2 and 5 µM). Samples (one biological replicate) were analyzed by western blot using an antibody against H3K27me3 (uncropped image). Total H3 protein levels were also assessed and no major variations were found. Ezh2 protein levels were also measured and no major variations were found. Tubulin levels are presented as loading control. This experiment was repeated twice.

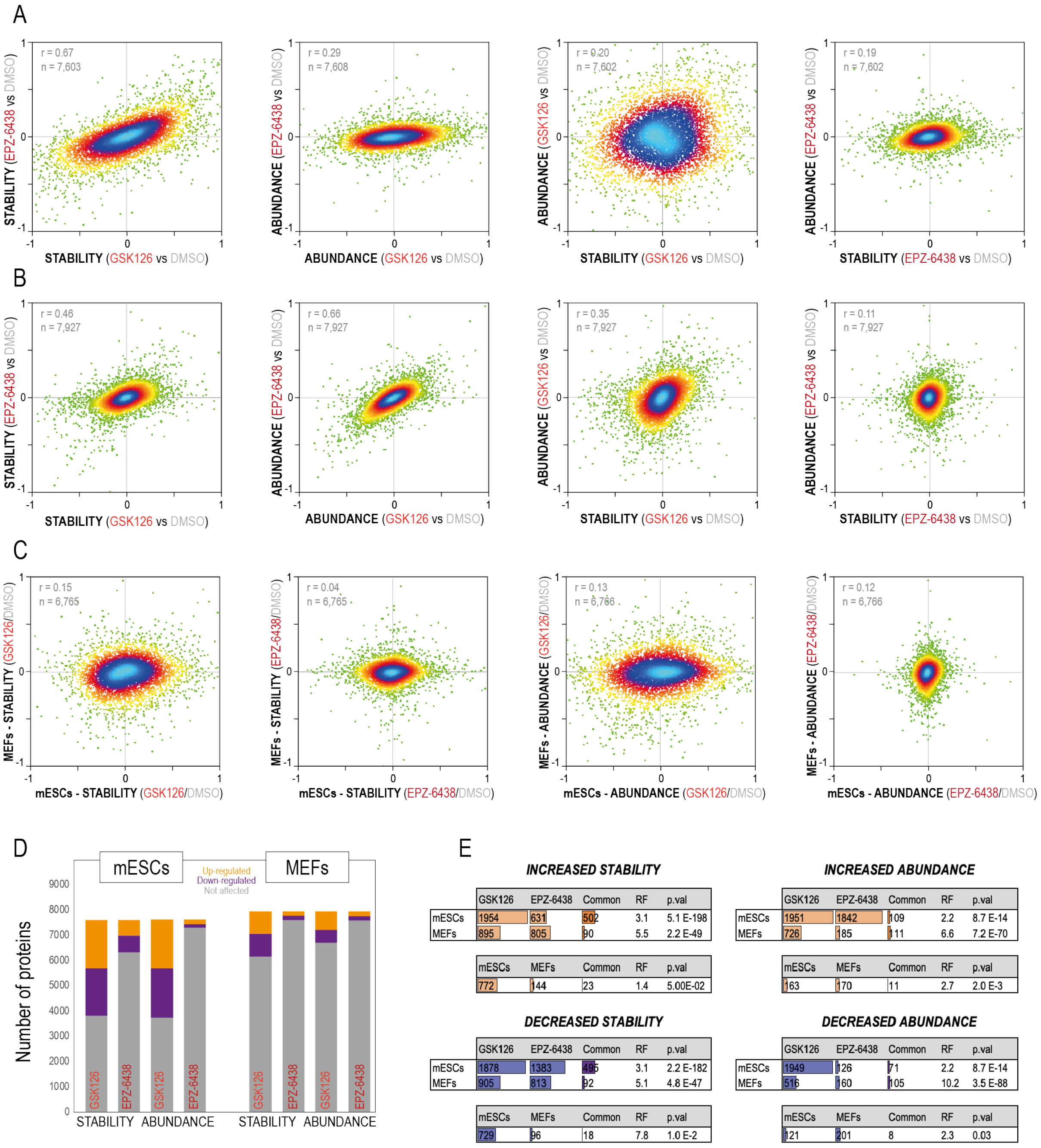

Supplementary Figure 13. Thermal stability and total protein abundance levels in mESCs and MEFs upon Ezh2 inhibition with two compounds. A, density scatterplots comparing stability and abundance changes in mESCs. B, density scatterplots comparing stability and abundance changes in MEFs. A, density scatterplots comparing stability and abundance changes between mESCs and MEFs. D, number of up- and down-regulated proteins (moderated t-test *limma*, FDR <5%) found in each comparison. E, number of proteins that were found regulated in common between both compounds (up) and between mESCs and MEFs (down) for stability and abundance changes. The significance of the overlap in each comparison was calculated with a hypergeometric test (two-sided). RF, representation factor. Source data are provided as a Source Data file.

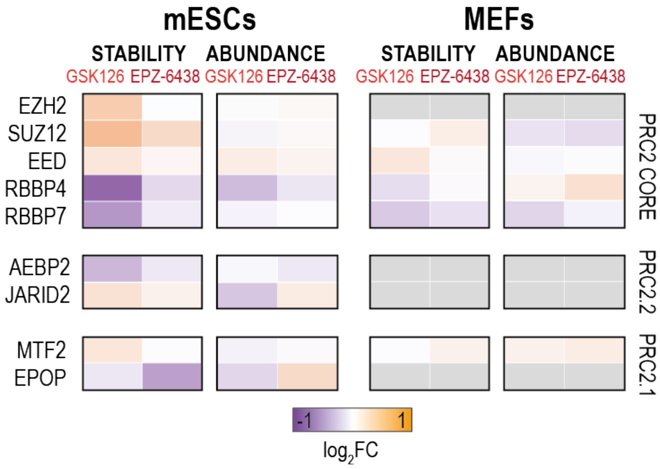

Supplementary Figure 14. Thermal stability and abundance changes of PRC2 complex upon Ezh2 inhibition. Heatmap showing stability and abundance changes in response to two different Ezh2 inhibitors (GSK126 and EPZ-6438) for all identified PRC2 subunits in both mESCs and MEFs. Proteins not identified are shown in grew.

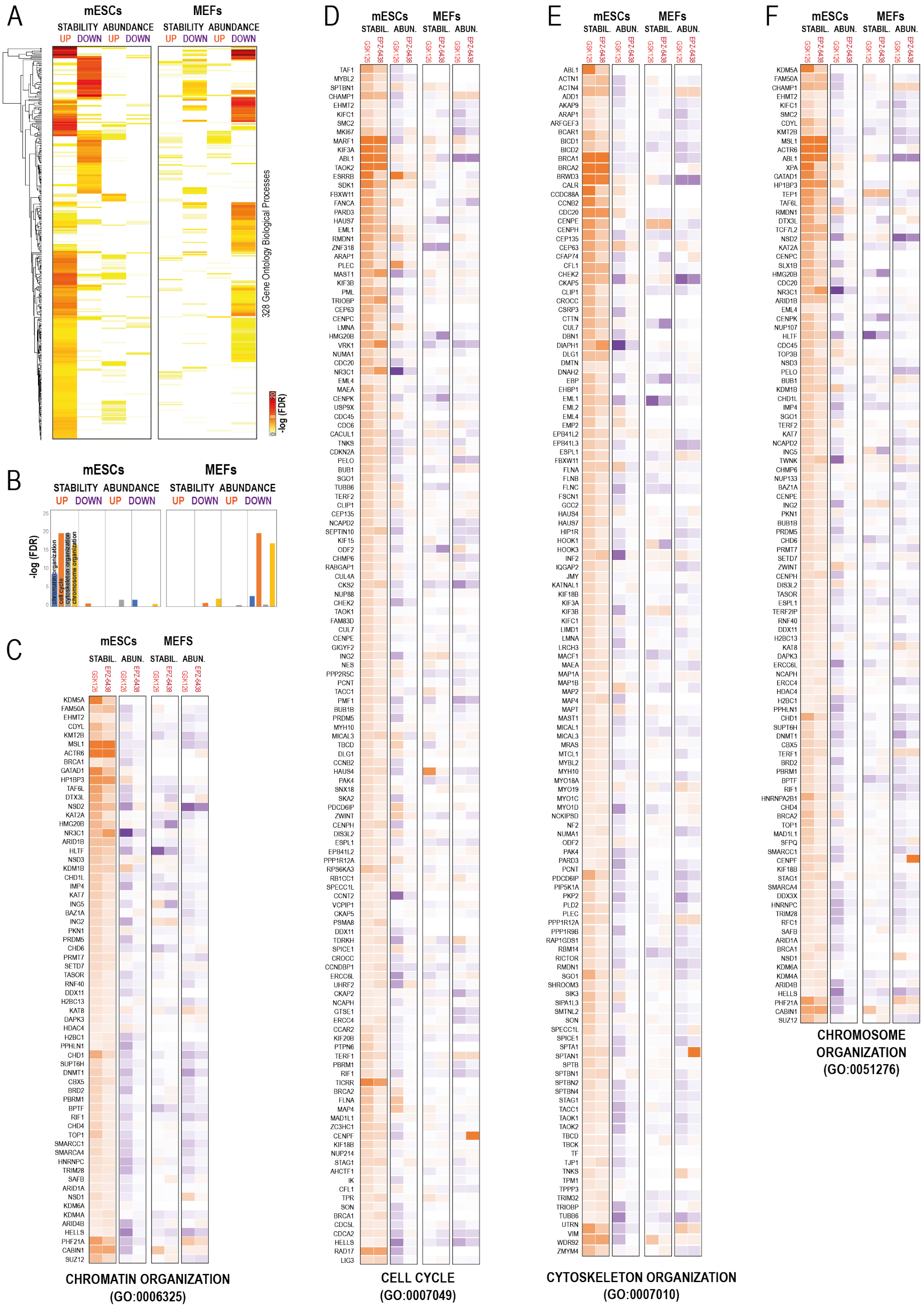

Supplementary Figure 15. Biological processes affected in mESCs and MEFs upon Ezh2 inhibition. A, heatmap showing all the biological processes (GO) that are enriched for proteins that showed differences in their stability or abundance levels in response to Ezh2 inhibitors in mESCs and MEFs. B, bar chart showing four terms that are enriched amongst the stabilized proteins in mESCs which are mentioned in the main text. C-F, heatmaps showing the actual stability and abundance changes of all the proteins that are annotated in these four GO terms.

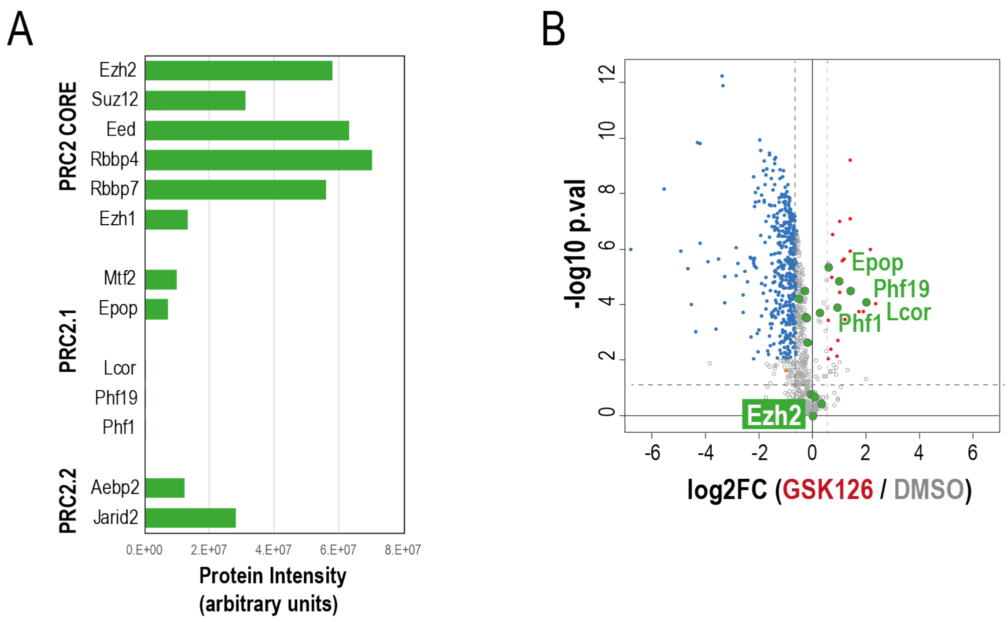

Supplementary Figure 16. The interactome of Ezh2 in mESCs in response to Ezh2 inhibition. A, protein intensity levels (arbitrary units) for all PRC2 subunits as well as proteins members of PRC2.1 and PRC2.2. subcomplexes. The observed stoichiometries are in agreement with previous reports[5]. B, volcano plot showing the differences in abundance for Ezh2 interactors in response to the Ezh2 inhibitor GSK126. Data was normalized by Ezh2 levels. Proteins that increased or decreased their abundances are shown in red and blue respectively. PRC2 complex members are shown in green. Source data are provided as a Source Data file.

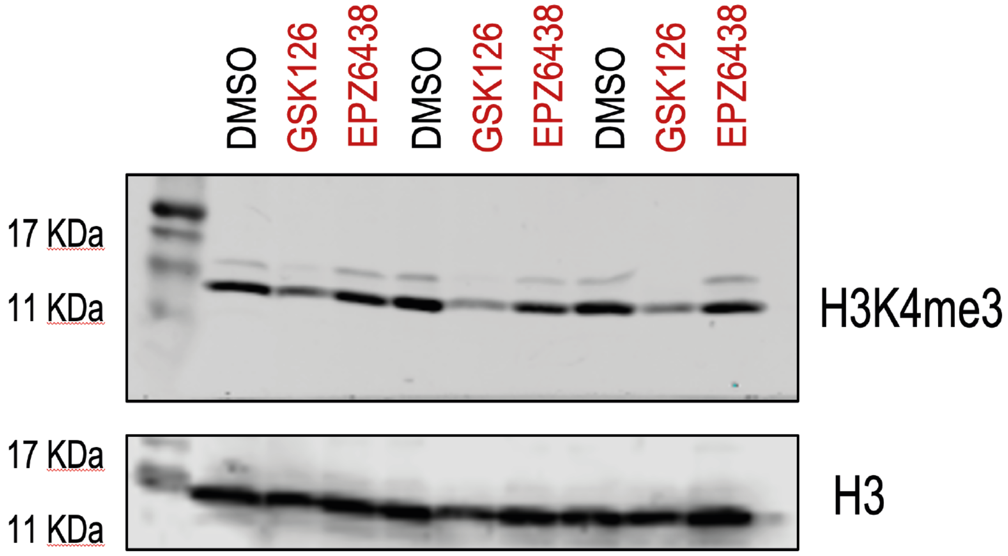

Supplementary Figure 17. H3K4me3 levels in mESCs in response to Ezh2 inhibition. mESCs were treated with GSK126 and EPZ6438 for two days and the levels of H3K4me3 were assessed by western blot. Total H3 levels are presented as a loading control. This experiment includes three independent biological replicates but was performed only once.

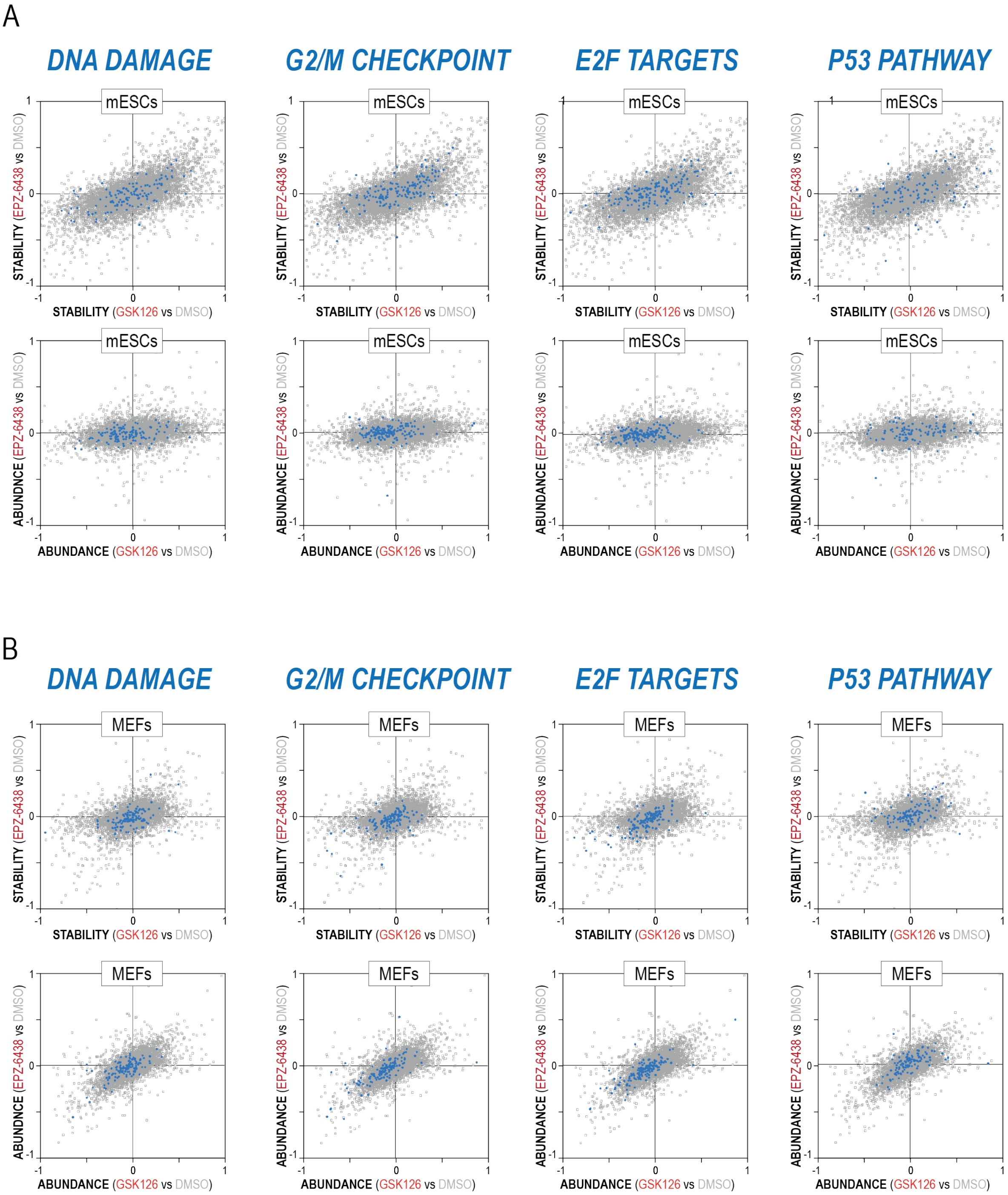

Supplementary Figure 18. Stability and abundance changes for several pathways related to DNA damage in response to Ezh2 inhibition. Proteins involved in DNA damage, G2/M checkpoint, E2F targets and p53 pathway were retrieved from the Hallmarks gene set at the Molecular Signature Database and are displayed in blue in the scatterplots. Stability (upper plots) and abundance (bottom plots) are shown for mESCs (A) and MEFs (B).

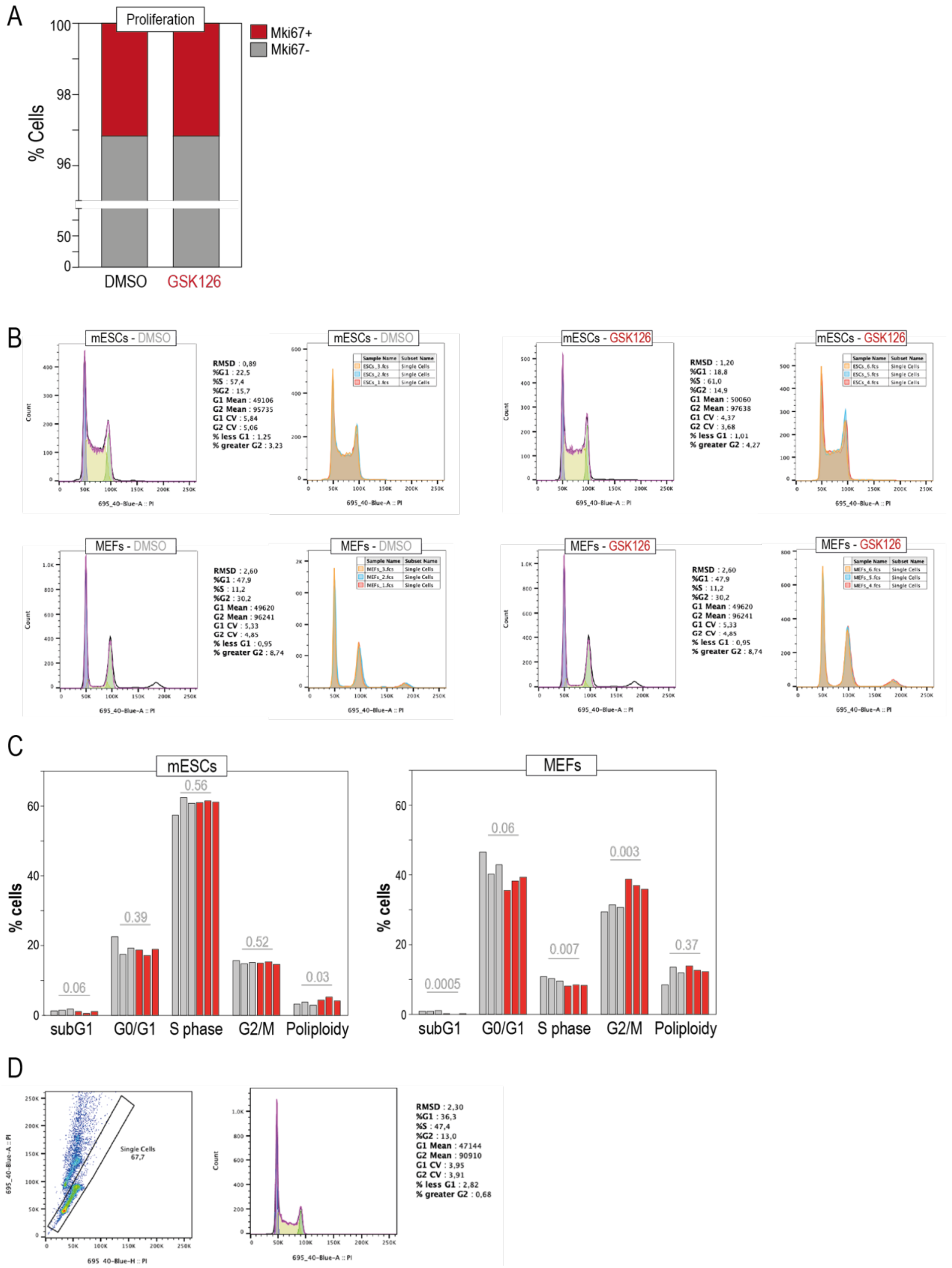

Supplementary Figure 19. Proliferation and cell cycle analysis. A, cell proliferation was determined by Mki67-positive cells in mESCs treated with GSK126 compared to control cells. (N=12,000 cells). B, cell cycle analysis by flow cytometry of mESCs and MEFs treated with either DMSO or GSK126. (N=3 experiments). C, quantification of cell cycle stages. P-values were calculated using a t-test (two-sided). D, Gating strategy DNA content analysis, cell aggregates were removed using pulse processing of the height versus area signals of the PI staining

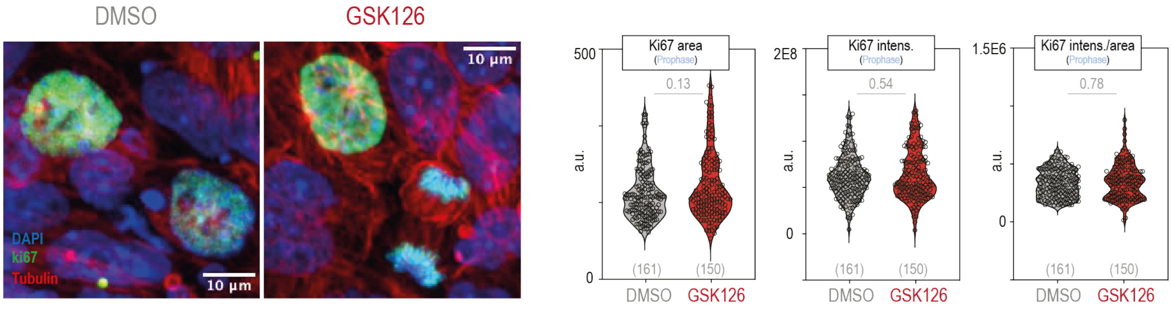

Supplementary Figure 20. Analysis of prophases in mESCs treated with GSK126. Examples of prophases in DMSO- and GSK126- treated mESCs (left). Chromosome area in prophases was determined using Ki67 (right). Ki67 intensity showed no differences in Ki67 recruitment to chromosomes during prophase in response to GSK126. P-values were calculated with a Mann Whitney U test (two-sided). Sample sizes are shown below in parenthesis. Source data are provided as a Source Data file.

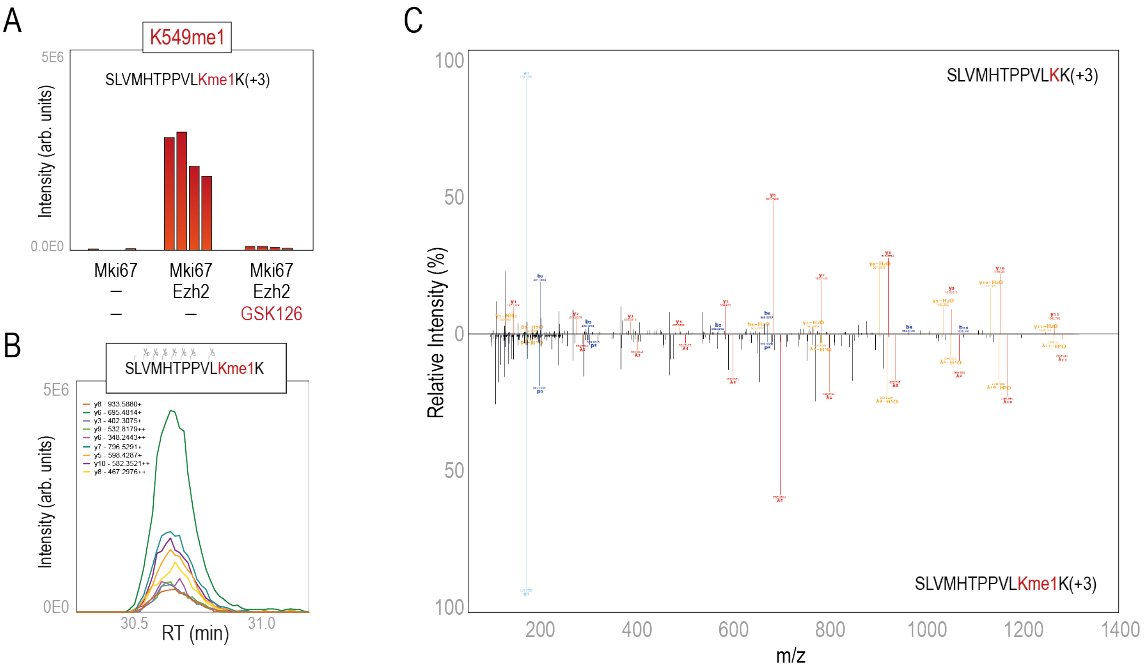

Supplementary Figure 21. *In vitro* mono-methylation of Mki67 in K549 by Ezh2. A, Quantification of the indicated Mki67 methylated peptide by PRM in an *in vitro* methyl-transferase assay using Ezh2. (arb. units, arbitrary units) Two biological replicates and two technical replicates were assayed. B, Integration of extracted ion chromatogram in Skyline showing the fragment ions used for quantification. C, comparison of MS/MS spectra from the methylated peptide and its unmodified counterpart.

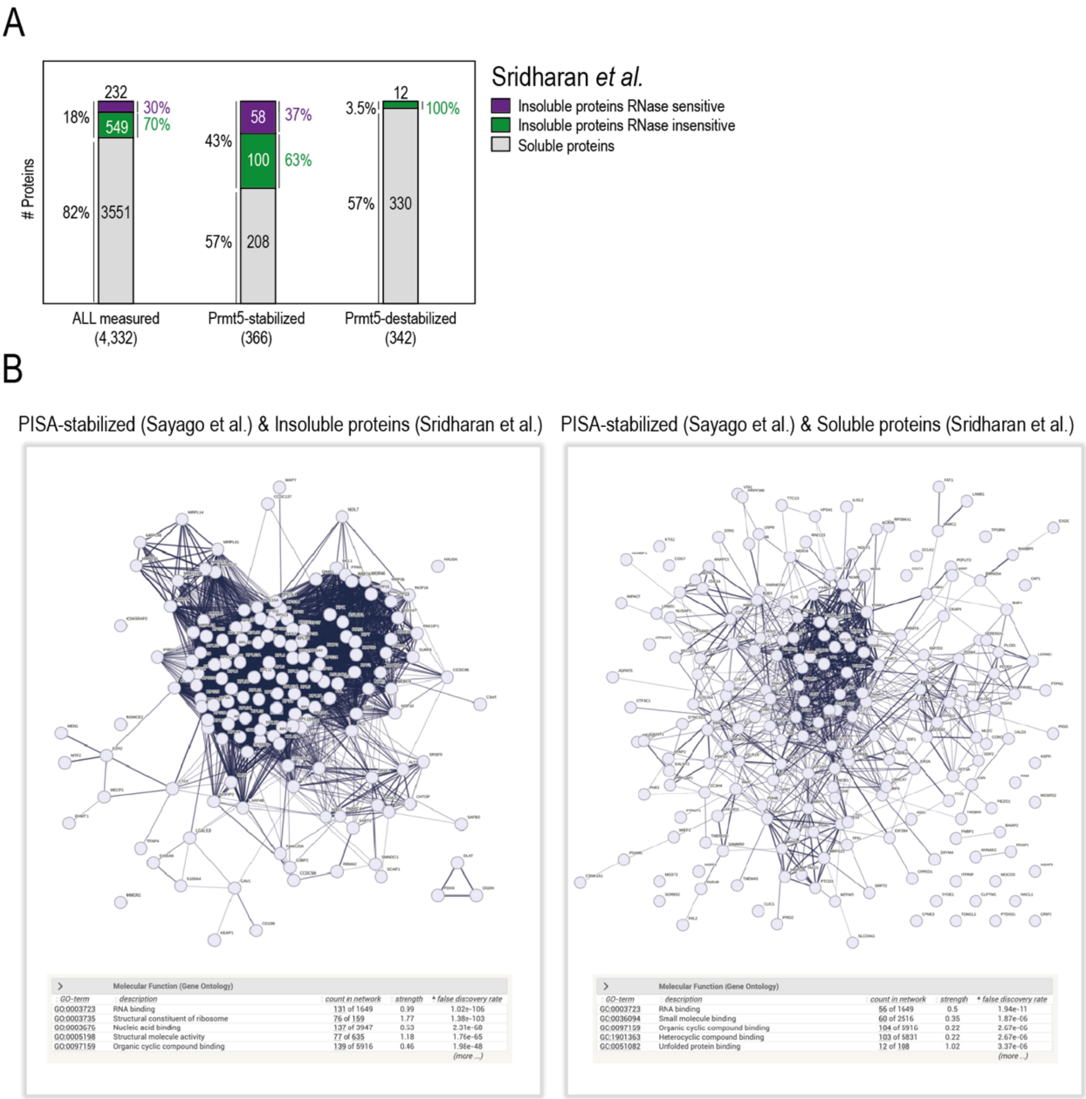

Supplementary Figure 22. Analysis of protein stability and protein solubility upon Prmt5 inhibition in mESCs. A, Proteins identified as soluble and insoluble were retrieved from Sridharan *et al.*[6]. Out of the 8,104 proteins assessed by PISA in our Prmt5 study, we were able to obtain solubility profiles for 4,322 of them. These 4,322 proteins showed no biases in the proportion of soluble (82%), insoluble RNA-sensitive (5%) and insoluble RNA-insensitive (12%) proteins as determined by Sridharan. However, the partition of soluble and insoluble proteins was markedly different between PISA-stabilized and destabilized proteins: 43% of the PISA-stabilized proteins were insoluble, a 2-fold increase compared to all measured (18%) proteins. B, GO enrichment analyses showed a significant enrichment in RNA binding proteins in both insoluble (left) and soluble (right) proteins that were found stabilized in our data.

SUPPLEMENTARY REFERENCES

1. Larsen, S. C. *et al.* Proteome-wide analysis of arginine monomethylation reveals widespread occurrence in human cells. *Sci. Signal.* **9**, rs9–rs9 (2016).
2. Chan-Penebre, E. *et al.* A selective inhibitor of PRMT5 with in vivo and in vitro potency in MCL models. *Nat. Chem. Biol.* **11**, 432–7 (2015).
3. Marmor-Kollet, H. *et al.* Spatiotemporal Proteomic Analysis of Stress Granule Disassembly Using APEX Reveals Regulation by SUMOylation and Links to ALS Pathogenesis. *Mol. Cell* **80**, 876-891.e6 (2020).
4. Youn, J. Y. *et al.* High-Density Proximity Mapping Reveals the Subcellular Organization of mRNA-Associated Granules and Bodies. *Mol. Cell* **69**, 517-532.e11 (2018).
5. van Mierlo, G., Veenstra, G. J. C., Vermeulen, M. & Marks, H. The Complexity of PRC2 Subcomplexes. *Trends Cell Biol.* **29**, 660–671 (2019).
6. Sridharan, S. *et al.* Systematic discovery of biomolecular condensate-specific protein phosphorylation. *Nat. Chem. Biol.* (2022). doi:10.1038/s41589-022-01062-y
7. Lee, J. M. *et al.* EZH2 Generates a Methyl Degron that Is Recognized by the DCAF1/DDB1/CUL4 E3 Ubiquitin Ligase Complex. *Mol. Cell* **48**, 572–586 (2012).
8. Liu, H. *et al.* A method for systematic mapping of protein lysine methylation identifies functions for HP1β in DNA damage response. *Mol. Cell* **50**, 723–735 (2013).
