# Supplementary material for: Decoding protein methylation function with thermal stability analysis
Source: Nat Commun. 2023 May 25;14:3016. doi: 10.1038/s41467-023-38863-1 (PMC10212966; doi:10.1038/s41467-023-38863-1)
Supplement: Supplementary file 3 — Description of Additional Supplementary File [file 41467_2023_38863_MOESM3_ESM.pdf]

File Name: Supplementary Data 1
Description: Proteins identified in mESCs and MEFs. S1 Proteomes sheet: Results from MaxQuant containing all the proteins identified in mESCs and MEFs using trypsin. Protein intensities were normalized with the LOESS function in the software Prostar.

File Name: Supplementary Data 2
Description: Protein isoforms identified in mESCs and MEFs. S2 All IDs trypsin-chymo sheet: Results from MaxQuant containing all the protein isoforms identified in mESCs and MEFs resulting from the combined analysis of trypsin and chymotrypsin data sets using the Ensembl database. S2 RMTs-KMTs-KDMs: Detailed information for all the protein isoforms identified in arginine methyl-transferases, lysine methyl-transferases and lysine de-methylases genes. All the remaining sheets show comparisons between the experimental spectra and the predicted spectra (using Prosit) for those unique peptides identified in the protein isoforms.

File Name: Supplementary Data 3
Description: Quantification by Parallel Reaction Monitoring of methyl-transferases and de-methylases in mESCs and differentiated cell types. S3 PRM sheet: Monitored peptides were quantified with Skyline and their abundances were summarized to calculate total protein intensity values (log scale) in each sample. Differential analysis was performed with Prostar using a moderated t-test (limma) (two-sided). Data was normalized using the LOESS function and missing values (displayed in blue in the table) were imputed with the K-Nearest Neighbors (KNN) function.

File Name: Supplementary Data 4
Description: Methylated residues and proteins identified in mESCs and MEFs. S4 Modifications sheet: Results from MaxQuant containing all the Arg and Lys methylation modifications identified in mESCs and MEFs. Arg methylation modifications are classified in different types of motifs. The probability of a residue to be localized within a disordered region is estimated with the IUPRED and ANCHOR scores. The intensities of all modifications were normalized with the LOESS function in Prostar. Additional functional information is shown in yellow for all the reported modifications which was annotated with Perseus software. S4 Residues sheet: information collapsed at the level of residues (i.e. modified amino acids). S4 Proteins sheet: information collapsed at the level of methylated proteins.

File Name: Supplementary Data 5
Description: GO enrichment analysis of identified methylated proteins. S5 Arg methylation sheet: Over-representation of GO terms (complete biological processes) was done with PantherDB using as input all the identified Arg methylated proteins. P-values were calculated with the Fisher's Exact test. FDR was estimated for multiple testing correction. Only terms found over-represented are shown. S5 Lysine methylation sheet: Over-representation of GO terms (complete biological processes) was done with PantherDB using as input all the identified Lys methylated proteins. P-values were calculated with the Fisher's Exact test. FDR was estimated for multiple testing correction. Only terms found over-represented are shown.

File Name: Supplementary Data 6
Description: Motif analysis of Arg methylated residues. S6 Non canonical RG sheet: Motif analysis for Arg methylation sites classified as non-canonical RG was done using MoMo. S6 Non canonical RG sheet: Motif analysis for Arg methylation sites classified as non-RG was done using MoMo. In both cases, the minimum number of occurrences was set to 8 and the p-value threshold was 0.000001. P-values were calculated with the Fisher's Exact test (two-sided).

File Name: Supplementary Data 7
Description: Stability and Abundance changes in mESCs and MEFs in response to Prmt5 and Ezh2 inhibitors. S7 Stability and Abundance sheet: Protein identification and quantification was done with MaxQuant. For the analysis of the effect of individual inhibitors, proteins were considered regulated when the p.val<0.01 and log2FC < -0.1 or >0.1. To call a protein regulated with "both" inhibitors, a protein must be regulated with the above criteria in at least one inhibitor and in the other inhibitor the change must be log2FC < -0.1 or >0.1 and the p.val <0.05. P-values were calculated using limma (two-sided) and adjusted for multiple testing with Benjamini-Hochberg.

File Name: Supplementary Data 8
Description: GO enrichment analysis of proteins with altered thermal stability upon Prmt5 inhibition. S8 GO analysis sheet: Over-representation of GO terms (complete biological processes) was done with PantherDB using as input proteins with increased or decreased thermal stability in response to GSK595 or GSK591 in mESC. P-values were calculated with the Fisher's Exact test. FDR was estimated for multiple testing correction.

File Name: Supplementary Data 9
Description: GO enrichment analysis of proteins with altered thermal stability upon Ezh2 inhibition. S9 GO analysis sheet: Over-representation of GO terms (complete biological processes) was done with PantherDB using as input proteins with increased or decreased thermal stability or abundance in response to Ezh2 inhibitors in mESC and MEFs. P-values were calculated with the Fisher's Exact test. FDR was estimated for multiple testing correction.

File Name: Supplementary Data 10
Description: Analysis of Ezh2 interacting proteins in mESCs. S10 Interactors Analysis: Results from the statistical analysis (limma) using Prostar showing the enrichment ratios, and p-values compared to negative controls (IgG) for all the identified proteins in mESCs treated with DMSO or GSK126. S10 GSK126 vs DMSO Analysis: Results from the statistical analysis (limma) comparing the levels of Ezh2 interactors between mESCs treated with GSK126 and DMSO. Proteins with partially observed values (POV) were imputed with the SLSA function in Prostar and are shown in blue. Proteins with missing on entire condition (MEC) were imputed with the DET quantile function in Prostar and are shown in orange. P-values were calculated using limma (two-sided) and adjusted for multiple testing with Benjamini-Hochberg.
