# Supplementary material for: Decoding protein methylation function with thermal stability analysis
Source: Nat Commun. 2023 May 25;14:3016. doi: 10.1038/s41467-023-38863-1 (PMC10212966; doi:10.1038/s41467-023-38863-1)
Supplement: Supplementary file 14 — Reporting Summary [file 41467_2023_38863_MOESM14_ESM.pdf]

nature portfolio

Corresponding author(s): Javier Muñoz Peralta
Last updated by author(s): Apr 28, 2023

Reporting Summary

Nature Portfolio wishes to improve the reproducibility of the work that we publish. This form provides structure for consistency and transparency in reporting. For further information on Nature Portfolio policies, see our Editorial Policies and the Editorial Policy Checklist.

Statistics

For all statistical analyses, confirm that the following items are present in the figure legend, table legend, main text, or Methods section.

n/a	Confirmed	
☐	☒	The exact sample size (*n*) for each experimental group/condition, given as a discrete number and unit of measurement
☐	☒	A statement on whether measurements were taken from distinct samples or whether the same sample was measured repeatedly
☐	☒	The statistical test(s) used AND whether they are one- or two-sided<br>*Only common tests should be described solely by name; describe more complex techniques in the Methods section.*
☒	☐	A description of all covariates tested
☐	☒	A description of any assumptions or corrections, such as tests of normality and adjustment for multiple comparisons
☐	☒	A full description of the statistical parameters including central tendency (e.g. means) or other basic estimates (e.g. regression coefficient) AND variation (e.g. standard deviation) or associated estimates of uncertainty (e.g. confidence intervals)
☐	☒	For null hypothesis testing, the test statistic (e.g. *F*, *t*, *r*) with confidence intervals, effect sizes, degrees of freedom and *P* value noted<br>*Give P values as exact values whenever suitable.*
☒	☐	For Bayesian analysis, information on the choice of priors and Markov chain Monte Carlo settings
☒	☐	For hierarchical and complex designs, identification of the appropriate level for tests and full reporting of outcomes
☒	☐	Estimates of effect sizes (e.g. Cohen's *d*, Pearson's *r*), indicating how they were calculated

Our web collection on statistics for biologists contains articles on many of the points above.

Software and code

Policy information about availability of computer code

Data collection	Mass spectrometry data was adquired using a Q Exactive HF-X (Thermo) using Xcalibur (Tune v2.11.0.3006) (Thermo)
Data analysis	Raw data was processed with MaxQuant software v1.6.10.43. PRM data were analyzed with Skyline v20.2.0.343. Statistical analyses were performed with Prostar v1.18.5. Microscopy image analysis was done with Definens Developer XD v2.0 (Definiens) and ImageJ (v1.52a) software. Flow cytometry data was analyzed with FlowJo (v10). Enrichment analysis was done with GSEA v 4.0.2.

For manuscripts utilizing custom algorithms or software that are central to the research but not yet described in published literature, software must be made available to editors and reviewers. We strongly encourage code deposition in a community repository (e.g. GitHub). See the Nature Portfolio guidelines for submitting code & software for further information.

Data

Policy information about availability of data

All manuscripts must include a data availability statement. This statement should provide the following information, where applicable:

- Accession codes, unique identifiers, or web links for publicly available datasets
- A description of any restrictions on data availability
- For clinical datasets or third party data, please ensure that the statement adheres to our policy

The mass spectrometry proteomics data generated in this study have been deposited in the ProteomeXchange Consortium via the PRIDE partner repository under

accession code PXD038939 [https://proteomecentral.proteomexchange.org/cgi/GetDataset?ID=PXD038939]. Mass spectrometry data were searched against a Mus musculus database (UniProtKB, 21,982 sequences)

Human research participants

Policy information about studies involving human research participants and Sex and Gender in Research.

Reporting on sex and gender	n/a
Population characteristics	n/a
Recruitment	n/a
Ethics oversight	n/a

Note that full information on the approval of the study protocol must also be provided in the manuscript.

Field-specific reporting

Please select the one below that is the best fit for your research. If you are not sure, read the appropriate sections before making your selection.

☒ Life sciences ☐ Behavioural & social sciences ☐ Ecological, evolutionary & environmental sciences

For a reference copy of the document with all sections, see nature.com/documents/nr-reporting-summary-flat.pdf

Life sciences study design

All studies must disclose on these points even when the disclosure is negative.

Sample size	No sample size calculation was performed. Sample size was determined based on prior experience in TMT-coded quantitative proteomics workflows. For thermal stability and protein abundance analyses, 3 independent biological replicates were analysed. The number of replicates were chosen to obtain enough statistical power with the isobaric labelling approach employed here, and is based on previous results (PMID: 27452035). In addition, two different inhibitors of Prmt5 and Ezh2 were used to determine common and, hence, robust alterations.
Data exclusions	No data were excluded in this study
Replication	All experiments included in the manuscript were replicated. For the quantification of methyl-transferases and de-methylases: two biological replicates from four different cell lines and mouse tissues were analysed by PRM. For the Proteome Integral Solubility Assay (PISA): three biological replicates were analysed. For the thermal stability analysis of G3bp2 (methylated vs non-methylated): two biological replicates were done. For the in vitro methyl-transferase assays of G3bp2 and Mki67: two biological replicates were performed. For the analysis of Ezh2 interactors: three biological replicates were performed. In all cases, significant differences were assessed using proper statistical tests which are described in the text and figure legends.
Randomization	In thermal stability analyses, no randomization was performed given that only two conditions were compared in each experiment, and those were TMT-labelled. For label-free experiments, each specific condition was run serially and biological replicates were interspersed to avoid technical biases and to avoid potential carry-over of "potentially positive conditions "over "negative controls". In the case of the methyl-transferase assays, in order to avoid potential carry-over of methylated peptides in the subsequent sample: negative control (substrate) were run first, followed by inhibitor-treated samples (substrate + enzyme + inhibitor) and the tested condition (substrate + enzyme) were run. In the case of the analysis of Ezh2 interactors, in order to avoid potential carry-over of interacting proteins over the negative control, the negative control samples (i.e. IgG) were run first, followed by GSK126-treated mESCs and, finally, mESCs. In all cases, blank samples were run between biological replicates, i.e. between sample series.
Blinding	In order to maintain a strict control and oversight over the sample preparation procedures, LC-MS/MS runs, and data analysis, the researchers did not use blinding when conducting the experiments reported in this work.

Reporting for specific materials, systems and methods

We require information from authors about some types of materials, experimental systems and methods used in many studies. Here, indicate whether each material, system or method listed is relevant to your study. If you are not sure if a list item applies to your research, read the appropriate section before selecting a response.

Materials & experimental systems

n/a	Involved in the study
☐	☒ Antibodies
☐	☒ Eukaryotic cell lines
☒	☐ Palaeontology and archaeology
☐	☒ Animals and other organisms
☒	☐ Clinical data
☒	☐ Dual use research of concern

Methods

n/a	Involved in the study
☒	☐ ChIP-seq
☒	☐ Flow cytometry
☒	☐ MRI-based neuroimaging

Antibodies

Antibodies used

Symmetric Di-Methyl Arginine Motif [sdme-RG] MultiMab™ Rabbit mAb mix #13222
https://www.cellsignal.com/products/primary-antibodies/symmetric-di-methyl-arginine-motif-sdme-rg-multimab-rabbit-mab-mix/13222
Dilution: 1:1000

Anti-SNRPD3 antibody produced in rabbit HPA001170 https://www.sigmaaldrich.com/GB/en/product/sigma/hpa001170?gclid=EAIaIQobChMIk4KbzJGk_QIVkNDtCh23qAfsEAAYAiAAEgLZP_D_BwE&gclsrc=aw.ds
Dilution: 1:1000

α-Tubulin (11H10) Rabbit mAb #2125
https://www.cellsignal.com/products/primary-antibodies/a-tubulin-11h10-rabbit-mab/2125
Dilution: 1:1000

Prmt5 mouse (A-11): sc-376937
https://www.scbt.com/p/prmt5-antibody-a-11?gclid=EAIaIQobChMIvKO7n5Kk_QIVTYBQBh0JMwZcEAAYASAAEgJFMPD_BwE
Dilution: 1:1000

Tri-Methyl-Histone H3 (Lys27) (C36B11) Rabbit mAb #9733
https://www.cellsignal.com/products/primary-antibodies/tri-methyl-histone-h3-lys27-c36b11-rabbit-mab/9733
Dilution: 1:1000

Histone H3 (D1H2) XP® Rabbit mAb #4499
https://www.cellsignal.com/products/primary-antibodies/histone-h3-d1h2-xp-rabbit-mab/4499
Dilution: 1:2000

Ezh2 (D2C9) XP® Rabbit mAb #5246
https://www.cellsignal.com/products/primary-antibodies/ezh2-d2c9-xp-rabbit-mab/5246
Dilution: 1:1000

Tri-Methyl-Histone H3 (Lys4) (C64G9) Rabbit mAb #9725
https://www.abcam.com/products/primary-antibodies/histone-h3-tri-methyl-k4-antibody-chip-grade-ab8580.html
Dilution: 1:1000

Goat anti-Rabbit IgG (H+L) Highly Cross-Adsorbed Secondary Antibody, Alexa Fluor™ 680
https://www.thermofisher.com/antibody/product/Goat-anti-Rabbit-IgG-H-L-Highly-Cross-Adsorbed-Secondary-Antibody-Polyclonal/A-21109
Dilution: 1:5000

Goat anti-Mouse IgG (H+L) Cross-Adsorbed Secondary Antibody, Alexa Fluor™ 680
https://www.thermofisher.com/antibody/product/Goat-anti-Mouse-IgG-H-L-Cross-Adsorbed-Secondary-Antibody-Polyclonal/A-21057
Dilution: 1:5000

Fibrillarin (C13C3) Rabbit mAb #2639
https://www.cellsignal.com/products/primary-antibodies/fibrillarin-c13c3-rabbit-mab/2639
Dilution: 1:200

eIF4E Monoclonal Antibody (5D11)
https://www.thermofisher.com/antibody/product/eIF4E-Antibody-clone-5D11-Monoclonal/MA1-089
Dilution: 1:200

Goat anti-Rabbit IgG (H+L) Highly Cross-Adsorbed Secondary Antibody, Alexa Fluor™ 568
https://www.thermofisher.com/antibody/product/Goat-anti-Rabbit-IgG-H-L-Highly-Cross-Adsorbed-Secondary-Antibody-Polyclonal/A-11036
Dilution: 1:200

Goat anti-Mouse IgG (H+L) Cross-Adsorbed Secondary Antibody, Alexa Fluor™ 488
https://www.thermofisher.com/antibody/product/Goat-anti-Mouse-IgG-H-L-Cross-Adsorbed-Secondary-Antibody-Polyclonal/

A-11001
Dilution: 1:200

Ki67 antibody [SP6] anti-rabbit:
https://www.abcam.com/ki67-antibody-sp6-ab16667.html
Dilution: 1:1000

α-Tubulin [DM1A] anti-mouse, T9026
https://www.sigmaaldrich.com/GB/en/product/sigma/t9026?gclid=EAIaIQobChMIwYGNkKik_QIVFJftCh1pNwq0EAAYAiAAEgKV4PD_BwE&gclsrc=aw.ds
Dilution: 1:1000

Goat anti-Rabbit IgG (H+L) Highly Cross-Adsorbed Secondary Antibody, Alexa Fluor™ 488
thermofisher.com/antibody/product/Goat-anti-Rabbit-IgG-H-L-Highly-Cross-Adsorbed-Secondary-Antibody-Polyclonal/A-11034
Dilution: 1:400

Donkey anti-Mouse IgG (H+L) Highly Cross-Adsorbed Secondary Antibody, Alexa Fluor™ 647
https://www.thermofisher.com/antibody/product/Donkey-anti-Mouse-IgG-H-L-Highly-Cross-Adsorbed-Secondary-Antibody-Polyclonal/A-31571
Dilution: 1:400

Validation

Symmetric Di-Methyl Arginine Motif [sdme-RG] MultiMab™ Rabbit mAb mix #13222
https://www.cellsignal.com/products/primary-antibodies/symmetric-di-methyl-arginine-motif-sdme-rg-multimab-rabbit-mab-mix/13222
81 citations (e.g. Nature chemical biology 11.6 (2015): 432-437. Nature Communications 13.1 (2022): 5676)

Anti-SNRPD3 antibody produced in rabbit HPA001170 https://www.sigmaaldrich.com/GB/en/product/sigma/hpa001170?gclid=EAIaIQobChMIk4KbzJGk_QIVkNDtCh23qAfsEAAYAiAAEgLZP_D_BwE&gclsrc=aw.ds
7 citations (e.g. Nature chemical biology 11.6 (2015): 432-437. Cell 135.3 (2008): 497-509.)

α-Tubulin (11H10) Rabbit mAb #2125
https://www.cellsignal.com/products/primary-antibodies/a-tubulin-11h10-rabbit-mab/2125
729 citations (e.g. Nature Communications 13.1 (2022): 6243. Nature communications 13.1 (2022): 3722.)

Prmt5 mouse (A-11): sc-376937
https://www.scbt.com/p/prmt5-antibody-a-11?gclid=EAIaIQobChMIvKO7n5Kk_QIVTYBQBh0JMwZcEAAYASAAEgJFMPD_BwE
32 citations (e.g. Nature communications 12.1 (2021): 3444. Molecular Carcinogenesis 60.7 (2021): 429-439)

Tri-Methyl-Histone H3 (Lys27) (C36B11) Rabbit mAb #9733
https://www.cellsignal.com/products/primary-antibodies/tri-methyl-histone-h3-lys27-c36b11-rabbit-mab/9733
1065 citations (e.g. Cell 125.2 (2006): 213-217. Nature 442.7098 (2006): 86-90.)

Histone H3 (D1H2) XP® Rabbit mAb #4499
https://www.cellsignal.com/products/primary-antibodies/histone-h3-d1h2-xp-rabbit-mab/4499
1418 citations (e.g. Oncotarget 8.2 (2017): 3396. Cancer Research 79.8 (2019): 2009-2020.)

Ezh2 (D2C9) XP® Rabbit mAb #5246
https://www.cellsignal.com/products/primary-antibodies/ezh2-d2c9-xp-rabbit-mab/5246
661 citations (e.g. Nature Communications 13.1 (2022): 6781. Nature Communications 13.1 (2022): 4199.)

Tri-Methyl-Histone H3 (Lys4) (C64G9) Rabbit mAb #9725
https://www.abcam.com/products/primary-antibodies/histone-h3-tri-methyl-k4-antibody-chip-grade-ab8580.html
1741 citations (e.g. Journal of the Korean Society for Library and Information Science 48.3 (2014): 353-379)

Goat anti-Rabbit IgG (H+L) Highly Cross-Adsorbed Secondary Antibody, Alexa Fluor™ 680
https://www.thermofisher.com/antibody/product/Goat-anti-Rabbit-IgG-H-L-Highly-Cross-Adsorbed-Secondary-Antibody-Polyclonal/A-21109
274 citations (e.g. Pflügers Archiv-European Journal of Physiology 475.5 (2023): 607-620)

Goat anti-Mouse IgG (H+L) Cross-Adsorbed Secondary Antibody, Alexa Fluor™ 680
https://www.thermofisher.com/antibody/product/Goat-anti-Mouse-IgG-H-L-Cross-Adsorbed-Secondary-Antibody-Polyclonal/A-21057
313 citations (PMID: 36894123)

Fibrillarin (C13C3) Rabbit mAb #2639
https://www.cellsignal.com/products/primary-antibodies/fibrillarin-c13c3-rabbit-mab/2639
78 citations (e.g. Cell, 186(1), 80-97)

eIF4E Monoclonal Antibody (5D11)
https://www.thermofisher.com/antibody/product/eIF4E-Antibody-clone-5D11-Monoclonal/MA1-089
7 citations (e.g. Biology of Reproduction 98.1 (2018): 102-114)

Goat anti-Rabbit IgG (H+L) Highly Cross-Adsorbed Secondary Antibody, Alexa Fluor™ 568
https://www.thermofisher.com/antibody/product/Goat-anti-Rabbit-IgG-H-L-Highly-Cross-Adsorbed-Secondary-Antibody-Polyclonal/A-11036

1702 citations (e.g. Brain Structure and Function (2023): 1-14)

Goat anti-Mouse IgG (H+L) Cross-Adsorbed Secondary Antibody, Alexa Fluor™ 488
https://www.thermofisher.com/antibody/product/Goat-anti-Mouse-IgG-H-L-Cross-Adsorbed-Secondary-Antibody-Polyclonal/A-11001
7003 citations (e.g. Emerging Microbes & Infections (2023): 2195020)

Ki67 antibody [SP6] anti-rabbit:
https://www.abcam.com/ki67-antibody-sp6-ab16667.html
1744 citations (e.g. Nature protocols 16.1 (2021): 239-262. Cellular Signalling 77 (2021): 109818.)

α-Tubulin [DM1A] anti-mouse, T9026
https://www.sigmaaldrich.com/GB/en/product/sigma/t9026?gclid=EAIaIQobChMIwYGNkKik_QIVFJftCh1pNwq0EAAYAiAAEgKV4PD_BwE&gclsrc=aw.ds
4793 citations (e.g. Neuropsychopharmacology 43.8 (2018): 1779-1788. Cell Cycle 18.4 (2019): 467-475.)

Goat anti-Rabbit IgG (H+L) Highly Cross-Adsorbed Secondary Antibody, Alexa Fluor™ 488
thermofisher.com/antibody/product/Goat-anti-Rabbit-IgG-H-L-Highly-Cross-Adsorbed-Secondary-Antibody-Polyclonal/A-11034
5964 citations (e.g. Biogerontology (2023): 1-14)

Donkey anti-Mouse IgG (H+L) Highly Cross-Adsorbed Secondary Antibody, Alexa Fluor™ 647
https://www.thermofisher.com/antibody/product/Donkey-anti-Mouse-IgG-H-L-Highly-Cross-Adsorbed-Secondary-Antibody-Polyclonal/A-31571
1591 citations (e.g. Nucleus 14.1 (2023): 2165602)

Eukaryotic cell lines

Policy information about cell lines and Sex and Gender in Research

Cell line source(s)

Mouse embryonic stem cell (ESCs) line G4 was obtained from the Transgenic Mouse Unit of CNIO from E3.5 mouse blastocysts (https://www.cellosaurus.org/CVCL_E222). All the other mESCs cell lines were obtained from Cyan Lynch and Manuel Serrano (IRB, Barcelona).
ES-E14TG2a (E14) is a blastocyst-derived, embryonic stem cell line that was isolated from the embryo of a mouse (https://www.cellosaurus.org/CVCL_9108). This is a derivative of one of several mouse embryonal stem cell (ES) lines developed by M. Hooper in 1987. V6.4 was obtained from mixed background C57BL6/129 blastocysts (https://www.cellosaurus.org/CVCL_6D29). F123 (male) was derived from a cross between Mus musculus strain 129/SV-Jae (129) and Mus castaneus (cast) (https://www.cellosaurus.org/CVCL_VC43).

Authentication

Pluripotency in all embryonic stem cell lines used in the study was assessed by the expression of key pluripotency factors.

Mycoplasma contamination

All cell cultures tested negative for mycoplasma.

Commonly misidentified lines
(See ICLAC register)

No commonly misidentified lines were used.

Animals and other research organisms

Policy information about studies involving animals; ARRIVE guidelines recommended for reporting animal research, and Sex and Gender in Research

Laboratory animals

Mus musculus, C57BL/6 background (8-10 weeks old)

Wild animals

Not used

Reporting on sex

To analyze the protein expression of methyl-transferases and de-methylases in differentiated cells, liver, lung and kidney tissues from two mice were obtained. The two mice used in this study were not disaggregated by sex, and both male and female were analysed. The protein expression of these enzymes in not sex-dependent.

Field-collected samples

no field-collected samples

Ethics oversight

All animal procedures carried out at the CNIO were performed according to protocols approved by the CNIO-ISCIII Ethics Committee for Research and Animal Welfare (CEIyBA) and the Autonomous Community of Madrid (CAM). Protocol IDs: PROEX285/15 and PROEX15/18

Note that full information on the approval of the study protocol must also be provided in the manuscript.
